# Supplementary material for: Dynamics of Borrelia burgdorferi-Specific Antibodies: Seroconversion and Seroreversion between Two Population-Based, Cross-Sectional Surveys among Adults in Germany
Source: Microorganisms. 2020 Nov 25;8(12):1859. doi: 10.3390/microorganisms8121859 (PMC7761102; doi:10.3390/microorganisms8121859)
Supplement: Supplementary file 1 [file microorganisms-08-01859-s001.pdf]

Supplementary Material

This supplementary material is supporting information alongside the article "Dynamics of *Borrelia burgdorferi* specific antibodies: Seroconversion and seroreversion between two population-based, cross-sectional surveys among adults in Germany", on behalf of the authors

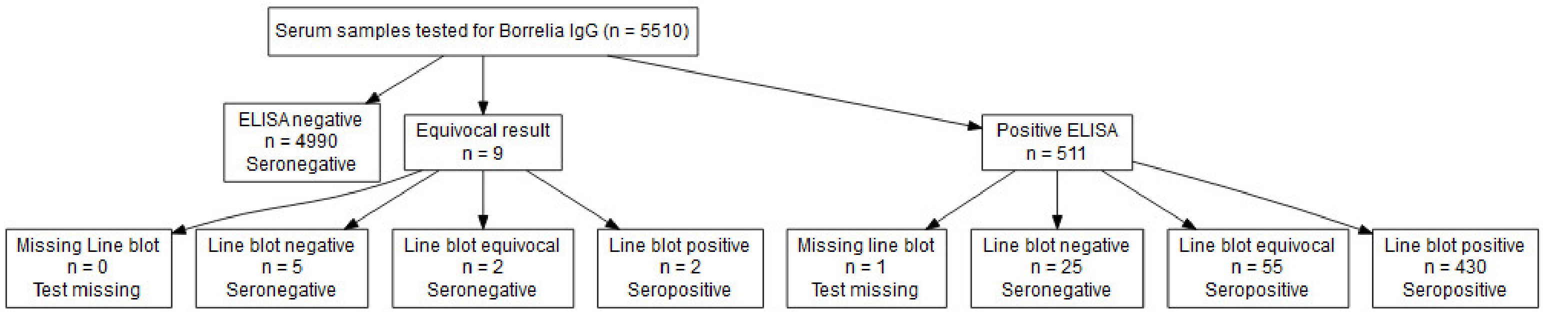

S. Figure 1. All samples of the population-based seroprevalence study in Germany, 1997-1999 were first tested with an ELISA to test for *B. burgdorferi* antibodies. In case of an equivocal or positive result, the *B. burgdorferi* antibodies were also assessed with an immunoblot.

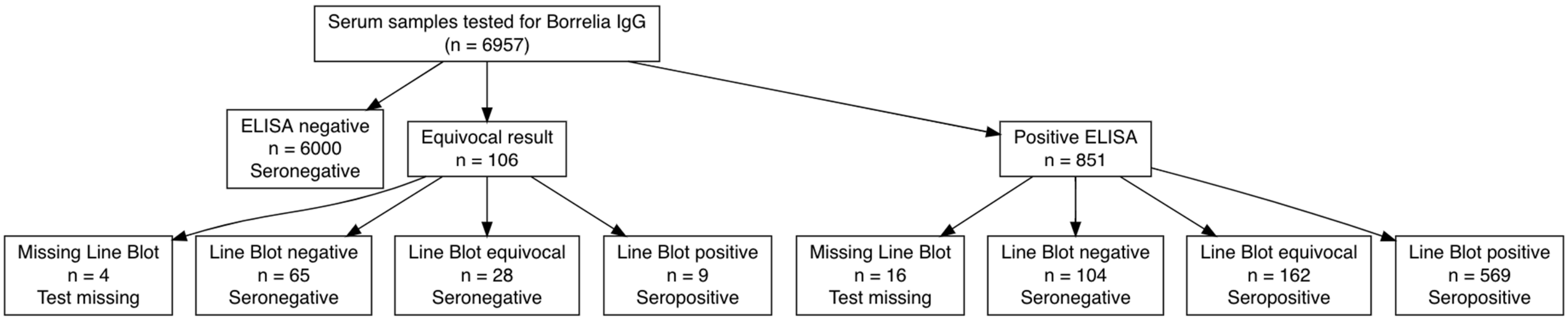

S. Figure 2. All samples of the population-based seroprevalence study in Germany, 2008-2011 were first tested with an ELISA to test for *B. burgdorferi* antibodies. In case of an equivocal or positive result, the *B. burgdorferi* antibodies were also assessed with an immunoblot.

Table 1 Estimated seroprevalence and 95% confidence intervals (CI) by several variables for two cross sectional population-based serosurveys in Germany, BGS98 in 1997-1999 (n = 5,510), and DEGS (n = 6,957) in 2008-2011.

	BGS98 (1997-1999)			*DEGS (2008-2011)*		
	Unweighted N	weighted Seroprevalence (%)	95% CI	Unweighted N	Seroprevalence (%)	95% CI
Sex						
Female	2757	6.27	4.99-7.55	3671	5.78	4.88-6.67
Male	2753	10.75	9.28-12.22	3406	12.94	11.28-14.60
Age group						
18-29	1047	5.11	3.56-6.66	1043	6.03	4.35-7.72
30-39	1278	3.94	2.78-5.11	827	6.29	4.04-8.53
40-49	1033	5.41	3.9-6.93	1269	6.33	4.72-7.93
50-59	1044	12.00	9.63-14.36	1374	8.53	6.62-10.45
60-69	728	17.39	14.22-20.56	1364	13.17	10.67-15.68
70-79	380	16.29	12.24-20.35	1080	20	16.68-23.32
*Place of residence**						
Baden-Württemberg	567	13.16	9.27-17.06	817	10.56	8.41-12.71
Bavaria	650	10.43	7.47-13.4	904	12.08	8.77-15.4
Central	712	9.64	6.53-12.74	886	10.41	7.71-13.1
Northwest	715	7.36	5.14-9.57	928	9.06	6.69-11.43
North Rhine-Westphalia	870	5.06	3.83-6.28	1299	5.33	3.68-6.97
East (north)	1052	7.24	5.78-8.69	1246	9.34	6.84-11.85
East (south)	944	9.57	7.49-11.66	997	11.88	8.94-14.82
Population						
< 5000	1262	12.18	10.08-14.28	1288	15.31	12.7-17.92
5000-<50,000	2105	8.57	6.97-10.16	3179	9.59	8.13-11.06
50,000-500,000	1397	8.06	5.83-10.28	1622	6.87	5.38-8.36
>500.000	746	4.89	3.45-6.32	988	6.94	4.97-8.91
Social economic status						
High	1178	8.51	6.74-10.28	1645	9.54	7.76-11.32
Middle	3338	8.70	7.47-9.93	4115	9.28	8.07-10.48
Low	865	8.00	5.82-10.18	1132	9.08	7.00-11.17

**Central comprises federal states Hessen, Rhineland-Palatinate, and Saarland; Northwest: Bremen, Hamburg, Lower Saxony, and Schleswig-Holstein; East (north): Berlin, Brandenburg, Mecklenburg-Vorpommern, and Saxony-Anhalt; East (south): Saxony, and Thuringia.*
